# Impact of Metal Salt Oxidants and Preparation Technology on Efficacy of Bacterial Cellulose/Polypyrrole Flexible Conductive Fiber Membranes

**DOI:** 10.3390/ma17061281

**Published:** 2024-03-10

**Authors:** Sixuan Tao, Qun Yang, Huili Qiu, Jie Zhu, Weimian Zhou, Juan Su, Ning Zhang, Lihui Xu, Hong Pan, Hongjuan Zhang, Jiping Wang

**Affiliations:** 1 School of Textiles and Fashion, Shanghai University of Engineering Science, Shanghai 201620, China; t736402112@163.com (S.T.); m390121217@sues.edu.cn (H.Q.); m390122203@sues.edu.cn (J.Z.); zhouweimian2022@163.com (W.Z.); m390123204@sues.edu.cn (J.S.); m390123203@sues.edu.cn (N.Z.); xulh0915@163.com (L.X.); 09180003@sues.edu.cn (H.P.); zhjdhdx@163.com (H.Z.); jpw@sues.edu.cn (J.W.); 2 Key Laboratory of Textile Fiber and Products (Wuhan Textile University), Ministry of Education, Wuhan 430200, China; 3 Shanghai Engineering Research Center for Clean Production of Textile Chemistry, Shanghai 201620, China

**Keywords:** bacterial cellulose, conductive membrane, polypyrrole, in situ polymerization, metal salt oxidants

## Abstract

In this study, we investigated the preparation and characterization of flexible conductive fiber membranes (BC/PPy) using different metal salt oxidants on bacterial cellulose (BC) and pyrrole (Py) in the in situ polymerization and co-blended methods, respectively. The effects of these oxidants, namely, ferric chloride hexahydrate (FeCl_3_·6H_2_O) and silver nitrate (AgNO_3_), on the structural characterization, conductivity, resistance value and thermal stability of the resulting materials were assessed by scanning electron microscopy (SEM), Fourier transform infrared (FTIR) spectroscopy, Raman spectroscopy and X-ray photoelectron spectroscopy (XPS). A comparative study revealed that the BC/PPy conductive fiber membrane prepared using FeCl_3_·6H_2_O as the oxidant had a resistance value of 12 Ω, while the BC/PPy conductive fiber membrane prepared using AgNO_3_ as the oxidant had an electrical resistance value of 130 Ω. The conductivity of the same molar ratio of BC/PPy prepared using FeCl_3_·6H_2_O as an oxidant was 10 times higher than that of the BC/PPy prepared using AgNO_3_ as an oxidant. Meanwhile, the resistance values of the conductive fiber membranes prepared from BC and PPy by the co-blended method were much higher than the BC/PPy prepared by in situ polymerization. SEM and XPS analyses revealed that when FeCl_3_·6H_2_O was used as the oxidant, the Fe-doped polypyrrole conductive particles could form uniform and dense conductive layers on the BC nanofiber surfaces. These two metal salt oxidants demonstrated differences in the binding sites between PPy and BC.

## 1. Introduction

Electronics are intimately integrated into our daily lives and distributed throughout the ambient environment, making it advantageous that they be freed from their rigid, confining encapsulation. This has had an impact on systems that use traditional metal wires to form fabric electrodes. With continued research, scholars have attempted to move away from the current fabric–metal-wire conductive systems, with many new materials currently under development, such as graphene [1], 2D carbon materials [2], MOFs [3], conductive polymers [4] and Mxenes [5].

MXenes consist of two-dimensional metal carbide and nitride materials with good flexibility and high conductivity. However, the conductivity of MXenes is closely related to the flake size, defects, doping and etching technology, with the preparation of a few/thin layers of highly conductive Mxenes considered difficult to scale up [6]. Conducting polymer composites such as polyaniline (PANI), polypyrrole (PPy) and polythiophene (PTh) offer a mature commercial scale, good biocompatibility [7] and excellent electrical conductivity [8], which are considered critical for the wide application of PPy composites. However, their low mechanical strength limits the application of PPy materials. To address the low mechanical strength of PPy materials, other materials with good film-forming properties, such as bacterial cellulose (BC) [9], polyvinyl alcohol (PVA) [10] and water-based polyurethane, have been introduced [11].

BC is a type of cellulose synthesized by microorganisms with high crystallinity and a high degree of polymerization. It is a polymer composed of β-D-glucose units linked by β-1,4-glycosidic bonds. These glycosidic linkages form a straight chain structure. Notably, BC exhibits a high molecular weight, with the degree of polymerization sometimes exceeding 10,000 [12]. The in situ polymerization and co-modification of polypyrrole/BC composites currently serve as the main preparation methods. In the presence of oxidizing agents, large numbers of hydroxyl groups will attach to monomer pyrrole through hydrogen bonds, forming PPy/BC composites. Blend modification can be achieved by adding the prepared PPy material to the BC substrate and filtering it into a film to obtain a composite material. However, this co-blended modification can provide a much lower conductivity compared to the composite obtained by in situ polymerization. The exploitation of the use of PPy materials has tended to focus on the electromagnetic shielding and thermal stability. Rani et al. discovered the preparation of PVA/aqueous polypyrrole (WPPY) composite films loaded with graphene quantum dots through solvent salivation [13]. However, the expensive WPPY only served to enhance the thermal stability of the film structure in the composite.

Halogens (I_2_, Br_2_), sulfuric acid (H_2_SO_4_) and ammonium persulfate ((NH_4_)_2_S_2_O_8_) have been previously used as oxidants to obtain PPy with low electrical conductivity, and metal salts have been used as pyrrole monomer oxidants to synthesize PPy with high electrical conductivity. The metal salts could complete both oxidation and doping in the reaction. Studying the influences of different oxidants on the material properties during PPy synthesis is an ongoing process. Saafan et al. investigated the effects of two different oxidizing agents, ferric chloride (FeCl_3_) and potassium persulfate (K_2_S_2_O_8_), on the dielectric properties of synthetic PPy [14]. Yussuf et al. built on previous studies and investigated the effects of FeCl_3_ and (NH_4_)_2_S_2_O_8_ on the electrical, thermal and morphological properties of PPy in aqueous systems containing the dopant sodium dodecyl sulfate [15]. In general, current research on oxidants has focused on a single PPy synthesis species, with a lack of research on the role of metal salt oxidants in the study of biomass/PPy composites. Moreover, studies on the effects of metal salt oxidants on biomass materials and the thermal stability properties of metal salt oxidants on membrane materials remain limited.

In this work, we formed BC/PPy composite membranes with electrical conductivity through the in situ polymerization of pyrrole (Py) and bacterial cellulose (BC) in aqueous solution and co-blended PPy with BC using different metal salt oxidants. The effects of the different metal salt oxidants and preparation technologies of the BC/PPy conductive fiber membranes on the electrical resistance value, thermal stability and microscopic morphology were subsequently investigated.

## 2. Materials and Methods

### 2.1. Materials

Bacterial cellulose (BC) homogenate, which was purchased from Guilin Qihong Technology Co., Ltd. (Guilin, China), was obtained after bacterial cellulose was purified by 1% sodium hydroxide (NaOH) and dispersed. The water content was up to 99.25%, the length of the BC fiber was 20 μm, the diameter was 50–100 nm, the crystal structure was cellulose type I and the surface functional group was -OH. Pyrrole (Py > 99.7%), silver nitrate (AgNO_3_), chloroform and ferric chloride hexahydrate (FeCl_3_·6H_2_O) were purchased from Sinopharm Chemical Reagent Co., Ltd. (Shanghai, China).

### 2.2. Preparation of BC/PPy Conductive Fiber Membrane

#### 2.2.1. BC/PPy Conductive Fiber Membrane Prepared by In Situ Polymerization

**Scheme 1:**
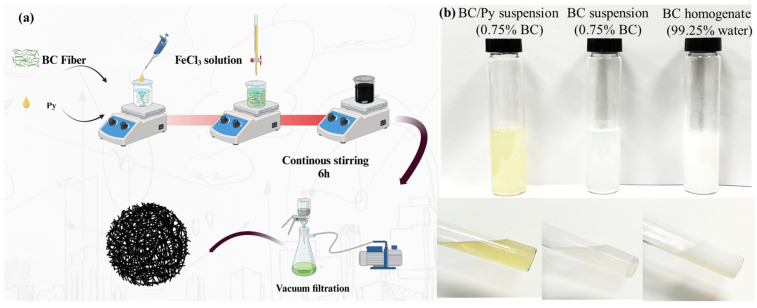
(**a**) BC/PPy fiber membranes prepared by in situ polymerization with different oxidants; (**b**) appearance and viscosity statuses of BC suspension (0.75% BC), BC homogenate (99.25% water) and BC/Py suspension (0.75% BC).

First, 5 g (0.75%) of BC homogenate was dispersed in 20 mL of deionized water, and then 0.4 mL of Py monomer was dissolved in the above liquid, followed by stirring (RCT Basic, IKA Digital, Staufen, German,) at 600 rpm for 30 min to mix the solution thoroughly. Then, 1.224 g of AgNO_3_ and 1.944 g of FeCl_3_·6H_2_O in 20 mL deionized water solutions were prepared as two different oxidant solutions. The oxidant solutions were then slowly dropped into the BC-Py liquid and polymerized at 200 rpm at room temperature for 6 h (Scheme 1). The prepared products were then labeled as BC/PPy-Fe and BC/PPy-Ag by vacuum filtration. The appearance and viscosity status of BC suspension (0.75% BC), BC homogenate (99.25% water) and BC/Py suspension (0.75% BC) can be seen in Scheme 1b. BC/Py suspension (0.75% BC) and BC suspension (0.75% BC) can be observed the lower viscosity than that of BC homogenate (99.25% water). After adding Py can not affect the viscosity of BC suspension (0.75% BC).

#### 2.2.2. BC/PPy Fiber Membrane Prepared by Co-Blended Method

**Scheme 2:**
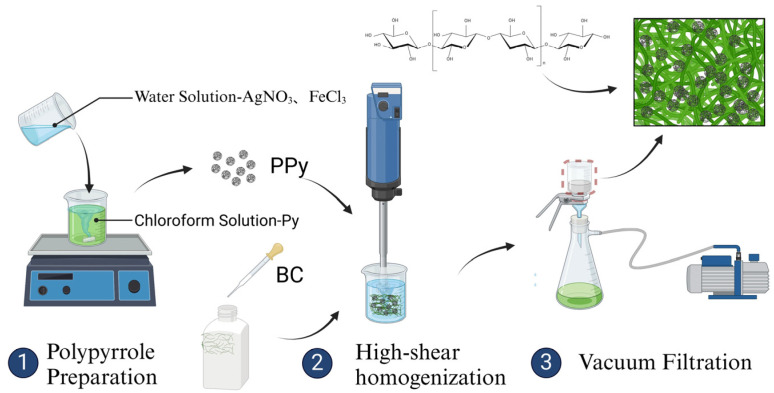
BC/PPy fiber membrane prepared by co-blended method with BC and PPy-Fe or PPy-Ag.

To obtain a more regular and highly polymerized PPy powder, polymerization was carried out by water/chloroform interfacial polymerization. Similarly, 1.224 g AgNO_3_ and 1.944 g FeCl_3_·6H_2_O were dissolved in 12 mL deionized water. Then, 0.4 mL Py monomer was dissolved in 18 mL chloroform. Because chloroform is insoluble in water, the water/chloroform interfaces remain stable. This stability is advantageous for the synthesis of PPy composites. After 12 h of interfacial polymerization, the resulting product was obtained through suction filtration. Subsequently, it was dried at 60 °C for 4 h in a vacuum oven [16,17]. The subsequent polymerized monomers were denoted as PPy-Fe and PPy-Ag and were polymerized at 200 rpm for 6 h at room temperature. We subsequently weighed 5 g (0.75%) of BC, which was dispersed in 20 mL of deionized water, and then weighed out 0.25 g each of PPy-Fe and PPy-Ag powder. After undergoing high-speed shearing (T25, IKA Digital, Staufen, Germany), the resulting products were extracted and filtered into films, which were denoted as BC/PPy-blending-Fe and BC/PPy-blending-Ag (Scheme 2).

### 2.3. Characterization

#### 2.3.1. FTIR Analysis

A Fourier transform infrared (FTIR) (Nicolet IS20, Thermo Scientific, Waltham, MA, USA) spectrometer was used to obtain the FTIR spectra with an attenuated total reflection pattern, with a scanning range of 400–4000 cm^–1^ and resolution of 4 cm^–1^. Each specimen was tested using a minimum of three different points.

#### 2.3.2. Thermogravimetric Analysis

Thermogravimetric analysis (TGA) is considered a suitable method for studying the thermal stability of composite materials. A Waters TG 55 analyzer was used for testing. Non-isothermal experiments were carried out at 10 °C/min from 20 °C to 600 °C, and the nitrogen flow was maintained constant at 40 mL. All materials were dried at 60 °C for 4 h in a vacuum oven.

#### 2.3.3. SEM Analysis

Micromorphology analysis of the PPy-Ag, PPy-Fe, BC/PPy-Ag and BC/PPy-Fe composites was conducted by a scanning electron microscope (SEM) (Hitachi Regulus8100, Hitachi, Tokyo, Japan) at 3 kV, and the microstructures of the BC/PPy-blending-Fe and BC/PPy-blending-Ag were also investigated by SEM. A 10 mA work current was used for sputtering the specimens by Quorum SC7620, (Hertfordshire, UK).

#### 2.3.4. XPS Analysis

X-ray photoelectron spectrometry (XPS) was used to characterize the surface compositions and doping degrees of the intrinsically conducting polymer and its composites. The parameters were set as follows: the excitation source was Al Kα radiation with a photon energy of 1486.6 eV, the beam size was 400 μm and the operating voltage was 12 kv.

#### 2.3.5. Raman Analysis

Raman spectroscopy (Horiba Scientific, Pairs, France) with an argon ion laser and an infrared spectrometer (Tokyo Instrument, INC, Tokyo, Japan) was employed to collect Raman spectra to determine the molecular structures. This work was conducted at an excitation wavelength of 633 nm.

#### 2.3.6. Resistance Testing

The electrical conductivities of the conductive membranes were measured using a 2010 multimeter (Keithley, Solon, OH, USA). Five different positions were selected to test and record the average resistance value.

#### 2.3.7. Tensile Strength Testing

Tensile strength testing was performed using a single-fiber strength tester. Rectangular strips (15 mm × 5 mm × ~0.25 mm) were cut from the dried specimens of the composite films. Three specimens per composite film were tested using a 10 N pressure gage at a tensile speed of 10 mm/min. The tensile strength and elastic modulus values of the specimens were calculated.

## 3. Results and Discussion

### 3.1. Micromorphology of BC/PPy Fiber Membranes

**Figure 1:**
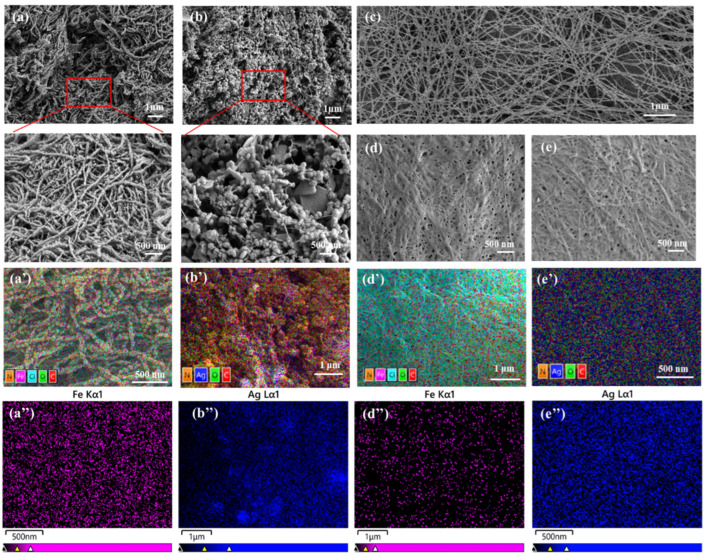
SEM images of (**a**) BC/PPy-Fe; (**b**) BC/PPy-Fe; (**c**) BC; (**d**) BC/PPy-blending-Fe; and (**e**) BC/PPy-blending-Ag. Mapping of (**a’**) BC/PPy-Fe; (**b’**) BC/PPy-Fe; (**d’**) BC/PPy-blending-Fe; and (**e’**) BC/PPy-blending-Ag. EDS of (**a’’**) Fe Kα1 of BC/PPy-Fe; (**b’’**) Ag Lα1 of BC/PPy-Fe; (**d’’**) Fe Kα1 of BC/PPy-blending-Fe; and (**e’’**) Ag Lα1 of BC/PPy-blending-Ag.

SEM is considered the most direct test method for observing a material’s micromorphology. Figure 1 shows the results of the SEM tests conducted on the BC/PPy-Fe (Figure 1a) and BC/PPy-Ag (Figure 1b) fiber membranes prepared using in situ polymerization and on the BC/PPy-blending-Fe (Figure 1d) and BC/PPy-blending-Ag (Figure 1e) prepared using the co-blended method, as well as on the BC (Figure 1c). Figure 1a’–e’ show the mapping of the BC/PPy fiber membranes.

From Figure 1a,a’, it can be observed that PPy was synthesized on the fibers of the BC and wrapped around the entire fiber to form a continuous conductive network for the BC/PPy-Fe fiber membrane compared to the macrostructure of the BC (Figure 1c). Although much PPy was synthesized on the fibers of the BC and wrapped around the fiber in the BC/PPy-Ag fiber membrane (Figure 1b), the conductive network was not as uniform as in the BC/PPy-Fe fiber membrane. Moreover, the clumping together of the PPy conductive particles in some places was also observed, as seen in Figure 1b. From Figure 1d,e, the results indicate that PPy was deposited between the BC fiber network in the form of conductive particles in the BC/PPy-blending-Fe and BC/PPy-blending-Ag composites prepared using the co-blended method. This suggested that the integrity of the conductive network constructed by the in situ polymerization method was superior to that constructed by the co-blended method. During the synthesis of the composites by in situ polymerization, the nanoparticles of both BC/PPy-Fe and BC/PPy-Ag were generated as Fe_2_O_3_, Fe_3_O_4_ and AgNPs. However, the AgNPs had a catalytic depolymerization effect on the BC fibers, which were not resistant to nitric acid. As a result, a large number of BC fibers depolymerized under the synergistic effect of the AgNPs and nitric acid, which were available for the monomeric Py adsorption sites, and so reduced the BC/PPy-Ag conductive network integrity.

### 3.2. Resistance of BC/PPy Conductive Fiber Membrane

The resistance values of the BC/PPy conductive fiber membranes were measured using a resistance test meter, and the results are shown in Table 1. A comparative study revealed that when using the same amount of BC and pyrrole monomers, the BC/PPy conductive fiber membrane prepared using FeCl_3_·6H_2_O as the oxidant had a resistance value of 12 Ω, while the BC/PPy conductive fiber membrane prepared using AgNO_3_ as the oxidant had an electrical resistance value of 130 Ω. The higher resistance value observed in the BC/PPy-Ag compared to BC/PPy-Fe can be attributed to the dependence of the PPy composite conductivity on dopant oxidation. In the experiment, the primary dopants were chloride (Cl^−^) and nitrate (NO_3_^−^). While NO_3_^−^ oxidation surpasses that of Cl^−^, excessive oxidation can lead to a reduction in the degree of PPy composite polymerization. Furthermore, stronger oxidation may break the glycosidic linkages in BC, inhibiting the formation of a complete conductive network, as evident in the SEM images (Figure 1a,b).

Meanwhile, the resistance values of the fiber membranes prepared from BC and PPy by the co-blended method were much higher than the BC/PPy prepared by in situ polymerization. BC/PPy-blending-Fe and BC/PPy-blending-Ag consisted of PPy-Fe and PPy-Ag powders, which were prepared by the interfacial method and dispersed between the BC fiber network through high-speed shearing. However, this failed because, during the homogeneous shearing and dispersion, the PPy powders were distributed between the BC fiber network in the form of particle packs, which was detrimental to conductivity. This conclusion is consistent with the observation result of the SEM. Therefore, the electrical conductivity of BC/PPy-Fe was superior to that of BC/PPy-Ag, and this was strongly related to the integrity of the BC fibers that were covered and wrapped by PPy and formed a continuous conductive network. Notably, the BC/PPy-Ag sample contained a large amount of AgNPs, which had a catalytic effect on the CO_2_ between the BC fibers. This made the BC/PPy-Ag conductive network significantly less complete than that of BC/PPy-Fe.

### 3.3. Chemical Structure of BC/PPy Conductive Fiber Membrane

**Figure 2:**
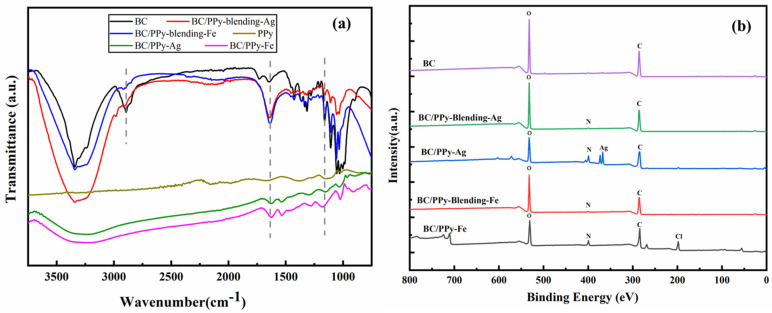
(**a**) FTIR spectra of BC; PPy; BC/PPy-Fe; BC/PPy-Ag; BC/PPy-blending-Fe; and BC/PPy-blending-Ag. (**b**) XPS spectra of BC/PPy-Ag; BC/PPy-Fe; BC; BC/PPy-blending-Fe; and BC/PPy-blending-Ag.

The infrared spectra of the BC/PPy-Fe, BC/PPy-Ag, BC/PPy-blending-Fe and BC/PPy-blending-Ag composites, as well as that of the BC, are shown in Figure 2a. The membrane materials exhibited a broad band at 1000–1060 cm^−1^, which likely came from the C-O and C-O-C groups. This was consistent with the literature [19], with 1528 cm^−1^ and 1436 cm^−1^ thought to be derived from the C-C and C-N vibrations of the standard pyrrole ring. This was consistent with the results obtained by Li et al. [20] in the literature. The membrane material had an additional absorption peak located at 1622 cm^−1^, which was similar to the 1645 cm^−1^ for the pure BC membrane material. The -O-H bending vibration peak of the absorbed water was determined, which was consistent with the results of Lay et al. [21]. The FTIR spectrum of the membrane prepared by the co-blended method was closer to that of pure BC. The polymerized PPy in the BC/PPy membrane prepared by the co-blended method did not generate hydrogen-bonding forces with the BC-rich hydrophilic groups and was only located in the form of particles in the gaps where the fibers were bound, which was detrimental to the formation of a conductive network.

To confirm the chemical states and elemental compositions of the composites, the total spectra obtained by XPS are shown in Figure 2b. The binding energies of the 284 eV, 399 eV and 530 eV peaks represented C1s, O1s and N1s, respectively. BC/PPy-Fe, however, contained two weaker peaks at 198 eV and 711 eV. BC/PPy-Ag had a peak at 367 eV, which corresponded to Ag3d. The four peaks at 283.9 eV, 284.6 eV, 286.4 eV and 288.5 eV corresponded to β-C in the pyrrole ring, C-H/C-C/C=C (α-C in the pyrrole ring) [22], C-N^+^ and C-O [23], respectively. The peak at 397.0 eV in the high-resolution energy spectrum of N1s belonged to the deprotonated N of Py (C=N^+^), while 398.15 eV corresponded to -N-H- in the pyrrole ring, and the characteristic peak at 400 eV came from C-N^+^, which was in agreement with the spectrum mentioned in the literature from the peak type to the value. This was critical for the analysis of Fe2p and O1s in the BC/PPy-Fe composites. The four peaks of 709.8 eV, 712.5 eV, 715.5 eV and 724.2 eV were obtained by a split-peak fitting operation for the Fe2p high-resolution energy spectrum, and they coincided with Fe^2+^2p3/2, Fe^3+^2p3/2, Fe^3+^2p1/2 and Fe2p^2+^1/2 21, respectively, which implied the coexistence of Fe^3+^ and Fe^2+^ in the BC/PPy-Fe with the presence of Fe_2_O_3_ and Fe_3_O_4_. The split-peak fitting operation of the high-resolution energy spectrum of the BC/PPy-Fe composite O1s clearly revealed that 529.7 eV, 531.3 eV and 532.8 eV corresponded to the O-H, C-O and surface-adsorbed oxygen of PPy, respectively [24]. This implied the presence of Fe_2_O_3_ and Fe_3_O_4_ on the BC fibers and the adsorption of Fe within the BC fibers. During the reaction, the BC fibers could produce active adsorption for the generated metal particles, the growth of PPy on the BC fibers was stable, and the constructed conductive network was more complete.

**Figure 3:**
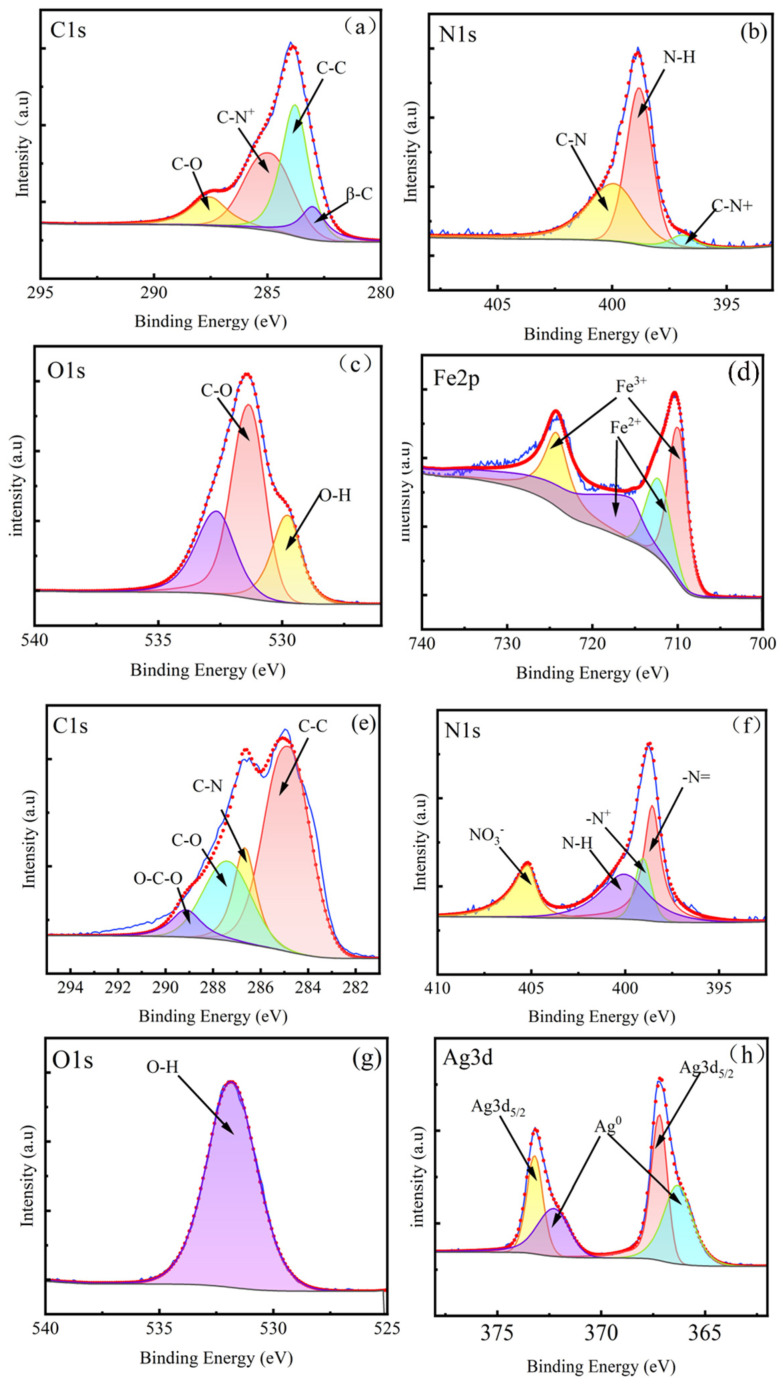
XPS: (**a**–**d**) BC/PPy-Fe (C, N, O, Fe) and (**e**–**h**) BC/PPy-Fe (C, N, O, Ag).

A split-peak fitting operation was performed on the C1s, N1s, O1s and Ag3d high-resolution spectra of the BC/PPy-Ag composite (Figure 3). The C1s spectra clearly showed binding energies (BEs) of 284.6 eV (C-C, C-H), 286.9 eV (C-OH) and 288.5 eV (O-C=O), with a new peak observed at 285.7 eV (C-N) [25]. The detailed chemical structure of the interfibrillar PPy was determined through a detailed analysis of the N1s profiles. The binding energies showed peaks at 398.5 eV, 398.7 eV, 396.3 eV and 405.5 eV, representing neutral benzenoid amine nitrogen (-NH-), protonated benzenoid amine nitrogen (-N-), quinonoid imine nitrogen (-N=) and nitrate ions (NO_3_^−1^), respectively [26]. According to the calculations of the PPy doping degree from Neoh et al., the percentage of area occupied by protonated aniline nitrogen (-N-) in the high-energy-resolved spectra of N1s after split-peak fitting was used as a doping degree measure [27,28]. The doping degree in the composite BC/PPy-Ag was 20.73%, which was close to that of nitrate (NO_3_^−^) (18.29%). Therefore, we approximated that all positive charges in the BC/PPy-Ag composite were balanced by nitrate (NO_3_^−^).

**Figure 4:**
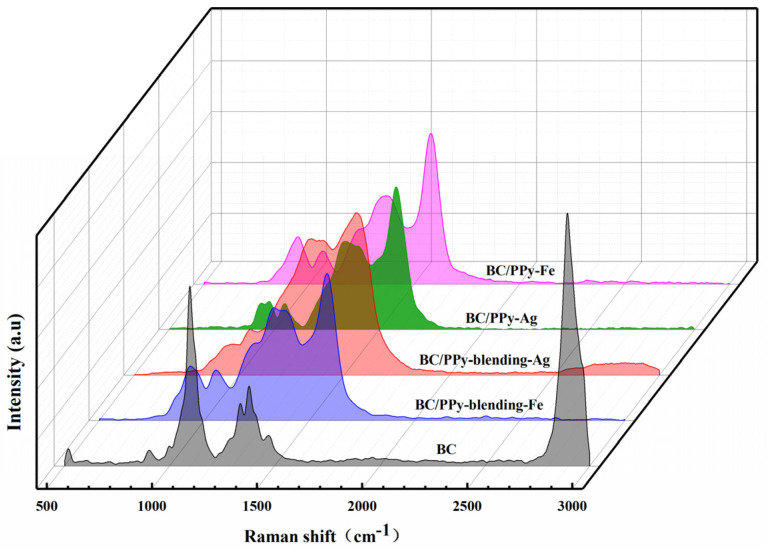
Raman spectra of BC; BC/PPy-Fe; BC/PPy-Ag; BC/PPy-blending-Fe; and BC/PPy-blending-Ag.

As shown in Figure 4, the BC/PPy-Ag, BC/PPy-Fe, BC/PPy-blending-Fe and BC/PPy-blending-Ag composites exhibited similar Raman bands. The main peak at 1582 cm^−1^ had some correlation with the C=C skeleton stretching, and the absorption peaks located at 1388 cm^−1^ and 1336 cm^−1^ [29] were thought to originate from the stretching vibration of the pyrrole ring [30]. The absorption peak at 1254 cm^−1^ was thought to belong to the in-plane deformation of -N-H-, with 1054 cm^−1^ and 940 cm^−1^ corresponding to the in-plane and out-of-plane deformations of -C-H-, respectively, and 968 cm^−1^ corresponding to the stretching mode of the -C-H-ring [31]. The conductivity of PPy was fundamentally dependent on the conjugation length of the polymer chains and the degree of oxidation of PPy in the system. The peaks at 1054 cm^−1^ and 940 cm^−1^ were related, respectively, to free radical cations and dications.

BC/PPy-blending-Fe had a ratio of 0.9 between polarons and bipolarons, while BC/PPy-blending-Ag had a ratio of 1.5. We used the ratio of the 1584 cm^−1^ of the C=C stretching vibration and the 1502 cm^−1^ of the C=C skeleton vibration to quantify the PPy polymer conjugation length. Surprisingly, this value was 1.74 for BC/PPy-blending-Fe and only 1.09 for BC/PPy-blending-Ag, indicating that the polymer conjugation length of BC/PPy-blending-Ag was much lower than that of BC/PPy-blending-Fe. To explore the intrinsic relationship between the ratio of free radical cations and dications of BC/PPy-blending-Fe and BC/PPy-blending-Ag and their conductivities, we further analyzed the peak intensities of BC/PPy-blending-Fe and BC/PPy-blending-Ag at 1054 cm^−1^ and 940 cm^−1^, respectively. The intensity of BC/PPy-blending-Ag at 1054 cm^−1^ was only 0.91 compared to BC/PPy-blending-Fe, while BC/PPy-blending-Ag at 940 cm^−1^ was 0.54 compared to BC/PPy-blending-Fe. This was due to the strong oxidizing property of NO_3_^−^ as a doping ion, which inhibited the polymer conjugation length and made the formation of long polymer chains difficult. The ratios of BC/PPy-Fe and BC/PPy-Ag at 1054 cm^−1^ and 940 cm^−1^ were 0.71 and 1.0, respectively, and the polymer conjugation lengths were 2.4 and 1.93, respectively. The increased polymer conjugation length improved the conductivity of BC/PPy-Fe and BC/PPy-Ag.

### 3.4. Thermal Properties of BC/PPy Conductive Fiber Membrane

**Figure 5:**
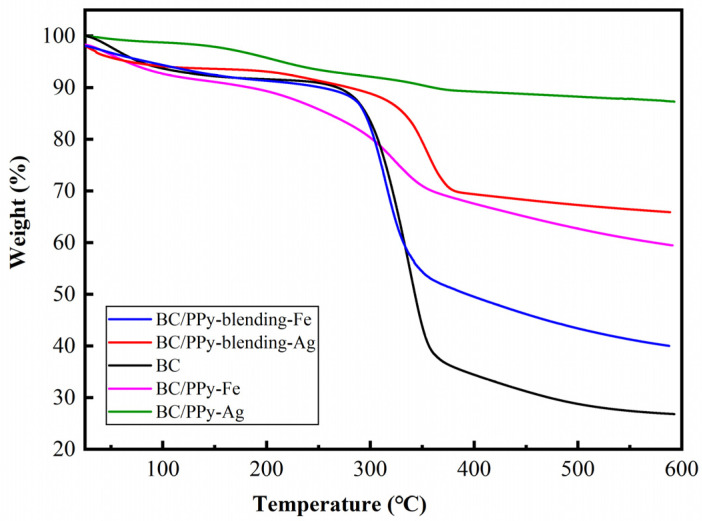
TGA curves of BC, BC/PPy-blending-Fe, BC/PPy-blending-Fe, BC/PPy-Fe and BC/PPy-Ag fiber membranes.

The TGA test is considered a widely accepted method for evaluating the thermal stability of a material. Prior to testing, the material was dried at 60 °C for 4 h in a vacuum oven to remove any free water from the material [16,17]. As shown in Figure 5, the TGA test divided the thermal weight loss into three regions. The weight loss in the region of 20–200 °C was mainly from the bound water between the BC fibers, which were not completely dried, and the not fully reacted pyrrole oligomers. The majority of the weight loss occurred in region II (200–400 °C), where the BC skeleton underwent disintegration for further weight loss, which was consistent with the literature [32]. The temperatures at which a 10% weight loss occurred for the BC/PPy-Fe, BC/PPy-Ag, BC/PPy-blending-Fe and BC/PPy-blending-Ag composites were 213.2 °C, 363.1 °C, 279.8 °C and 297.9 °C, respectively. When the temperature in region III was above 400 °C, weight loss mainly originated from the decomposition of the main chain. At the temperature of 600 °C, the residual carbon weights in the BC/PPy-Fe, BC/PPy-Ag, BC/PPy-blending-Fe and BC/PPy-blending-Ag composites were 60.9%, 87.4%, 41.7% and 66.9%, respectively. BC/PPy-Ag and BC/PPy-Fe obtained by in situ polymerization invariably showed a forward shift in the starting temperature. We attributed this phenomenon to the loss of some hydrogen bonds in the BC fiber network, due to the presence of the conducting polymer.

**Figure 6:**
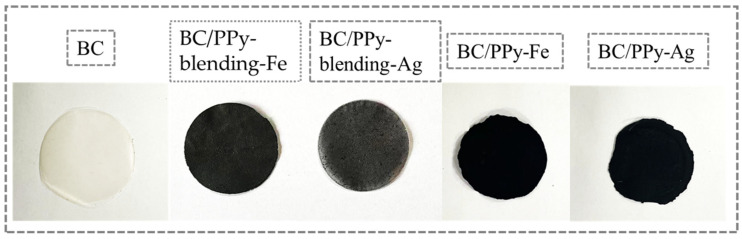
Macroscopic pictures of BC; BC/PPy-Fe; BC/PPy-Ag; BC/PPy-blending-Fe; and BC/PPy-blending-Ag.

Based on the data presented in Figure 5, BC/PPy-Fe exhibited the highest onset decomposition temperature compared to BC/PPy-Ag. Additionally, the weight loss region II of the BC/PPy-Fe fiber membrane, which was between 200 °C and 400 °C, was the same as that of the BC, indicating that the fiber membrane contained the component of BC. This phenomenon was also observed in the TGA curves of BC/PPy-blending-Fe and BC/PPy-blending-Fe. However, in the TGA curve of BC/PPy-Ag, the highest onset decomposition temperature was not observed, and the curve was relatively smooth. We attributed the observed phenomenon to the following two aspects: (1) the inevitable generation of silver nanoparticles (AgNPs) during the redox process using silver nitrate as the oxidizing agent, in which, as mentioned in the literature, the AgNPs will have a catalytic effect on the CO_2_ in the BC fiber network [33]; (2) the dissolution of silver nitrate in water, producing nitric acid, with the high nitric acid concentration producing hydrolysis in the amorphous and crystalline regions of the cellulose, further reducing the BC/PPy-Ag onset degradation temperature [34]. The higher residual carbon weight observed in the BC/PPy composite compared to the BC can be attributed to the abundant presence of metal nanoparticles within the composite materials. The increased quantity of metal nanoparticles directly contributes to elevated residual carbon contents. Specifically, Ag^+^ and Fe^3+^ serve as the primary active centers during polymerization. The Py monomers undergo redox reactions in the vicinity of these active centers. Additionally, a portion of the Py monomers become absorbed by BC. During the polymerization process, Ag^+^-Py, Fe^3+^ and BC-Py form interconnected molecular chains. Analyzing the TGA curve for BC/PPy-Ag, a gradual reduction in mass was observed. Its residual carbon weight at 600 °C was 87.4%. This phenomenon may be attributed to the strong oxidation of AgNO_3_, which could potentially disrupt the glycosidic linkages in BC or lead to its degradation [27,35]. An analysis of Figure 6 allowed us to draw the following conclusion, which was closely related to the above observations: during the synthesis of the in situ polymerized BC/PPy-Fe and BC/PPy-Ag, the Py monomer adsorbed the hydroxyl groups on the BC to form active sites, and a more complete conductive network was constructed on the BC fibers catalyzed by the oxidant. This conclusion is consistent with the observation results of the SEM in Figure 1 and the resistance values in Table 1.

### 3.5. Mechanical Properties of BC/PPy Conductive Fiber Membrane

The exceptional mechanical properties of pure BC membranes primarily arise from interfiber hydrogen-bonding forces. The tensile strengths and elongations at break were assessed using an electronic universal testing machine. The results are presented in Table 2, and macroscopic pictures of the BC, BC/PPy-Fe, BC/PPy-Ag, BC/PPy-blending-Fe and BC/PPy-blending-Ag are shown in Figure 6. Notably, the tensile strengths and elongations at break of the BC/PPy composites, prepared by in situ polymerization, were found to be lower than those of the BC/PPy-blending composites. This discrepancy can be attributed to the substantial consumption of hydroxyl groups during the in situ polymerization process, coupled with the oxidation of AgNO_3_ and FeCl_3_·6H_2_O. These oxidants have the potential to disrupt the glycosidic linkages within the BC structure, or to even lead to its degradation. Specifically, the tensile strengths for BC/PPy-Fe and BC/PPy-Ag were 6.94 ± 1.0 MPa and 5.78 ± 0.8 MPa, respectively, and the corresponding elongations at break were 4.66 ± 0.4% and 3.12 ± 0.3%, respectively. Interestingly, BC/PPy-Fe exhibited superior mechanical properties compared to BC/PPy-Ag. This difference can be attributed to the strong oxidation of AgNO_3_, which could contribute to BC degradation. Conversely, the tensile strengths for BC/PPy-blending-Fe and BC/PPy-blending-Ag were 16.7 ± 1.5 MPa and 16.1 ± 1.4 MPa, respectively, with elongations at break of 12.1 ± 0.5% and 11.7 ± 0.6%, respectively. The higher tensile strengths observed in the BC/PPy blending relative to the pure BC membrane can be explained by the increased friction forces between the BC and PPy filler.

## 4. Conclusions

In this study, the electrical resistance value of the flexible conductive material BC/PPy from 10 Ω to 22 kΩ depended on the utilized oxidant and preparation method. Using the co-blended method to prepare composites, PPy consisted of particles dispersed between the three-dimensional network of BC fibers. In situ polymerization was used to prepare BC/PPy flexible conductive fiber membranes. PPy was synthesized on the fibers of BC and wrapped around the entire fiber to form a continuous conductive network. The wrapped forms on the surfaces of BC fibers using different oxidants were microscopically different: PPy formed continuous conductive layers on the surfaces of the BC fibers when FeCl_3_·6H_2_O was used as the oxidant, and when AgNO_3_ was used as the oxidant, PPy formed sheet-like conductive layers on the surfaces of the BC fibers. Both AgNPs generated during polymerization using the AgNO_3_ oxidant and the dopant NO_3_^−^ had a catalytic depolymerization effect on the BC fibers; thus, the integrity of the BC/PPy-Ag conductive network was found to be much lower than that of the BC/PPy-Fe. We found that the type of oxidant and synthesis method were of crucial importance for the BC/PPy composites. This study demonstrated the promising potential for fabricating flexible, cost-effective and high-performance nanofibrous composite membranes suitable for biosensors. Moving forward, we will delve into the methods and mechanisms underlying the enhancement of the strength and flexibility in these composite films.

## Figures and Tables

**Table 1:** Resistances of BC/PPy-Fe, BC/PPy-Ag, BC/PPy-blending-Fe, BC/PPy-blending-Ag and BC, compared with the literature data.

Samples	Resistance (Ω)
BC/PPy-Fe	13
BC/PPy-Ag	130
BC/PPy-blending-Fe	5000
BC/PPy-blending-Ag	>22,000
BC	>22,000
BC-SnO_2_-PPy [18]	35.7

**Table 2:** Mechanical properties of BC/PPy-Fe; BC/PPy-Ag; BC/PPy-blending-Fe; BC/PPy-blending-Ag; and BC.

Samples	Tensile Strength (Mpa)	Elongation at Break (%)
BC/PPy-Fe	6.94 ± 1.0	4.66 ± 0.4
BC/PPy-Ag	5.78 ± 0.8	3.12 ± 0.3
BC/PPy-blending-Fe	16.7 ± 1.5	12.1 ± 0.5
BC/PPy-blending-Ag	16.1 ± 1.4	11.7 ± 0.6
BC	15.4 ± 1.3	14.0 ± 0.4

## Data Availability

Data are contained within the article.
